# Patient and prescriber perspectives on long-acting injectable (LAI) antipsychotics and analysis of in-office discussion regarding LAI treatment for schizophrenia

**DOI:** 10.1186/1471-244X-13-261

**Published:** 2013-10-16

**Authors:** Steven Potkin, Rimal Bera, Donna Zubek, Gina Lau

**Affiliations:** 1 Department of Psychiatry and Human Behavior, University of California, Irvine, School of Medicine, Irvine, California, 5251 California Avenue, Suite 240, Irvine, CA 92697-3960, USA; 2 School of Medicine, Irvine, University of California, Bldg 3-Rt 88, 101 The City Drive, Mail Code: 1680, Orange, CA 92668, USA; 3 Otsuka America Pharmaceutical Inc, 1 University Square Drive, Suite 500, Princeton, NJ 08540, USA

**Keywords:** Long-acting injectable antipsychotics, Patient perspectives, Prescriber perspectives, Schizophrenia, Treatment benefits, Community mental health, Office-visit discussions, Patient attitudes, Psychiatrists attitudes, Depot antipsychotics, Ethnographic

## Abstract

**Background:**

The research goal is to better understand prescriber, patient, and caregiver perspectives about long-acting injectable (LAI) antipsychotic therapy and how these perspectives affect LAI use. Addressing these perspectives in the clinic may lead to greater success in achieving therapeutic goals for the patient with schizophrenia.

**Methods:**

Ethnographic information was collected from a non-random sample of 69 prescriber-patient conversations (60 with community mental health center [CMHC] psychiatrists; 9 with nurse-practitioners) recorded during treatment visits from August 2011 to February 2012, transcribed and analyzed. Discussions were categorized according to 11 predetermined CMHC topics. In-person observations were also conducted at 4 CMHCs, including home visits by researchers (n = 15 patients) prior to the CMHC visit and observations of patients receiving injections and interacting with staff. Telephone in-depth interviews with psychiatrists, patients, and caregivers to gather additional information on LAI discussion, prescription, or use were conducted.

**Results:**

Antipsychotic treatment decisions were made without patient or caregiver input in 40 of 60 (67%) of psychiatrist-patient conversations. Involvement of patients or caregivers in treatment decisions was greater when discussing LAI (15 of 60 [25%]) vs oral antipsychotic treatment (5 of 60 [8%]). LAIs were not discussed by psychiatrists in 11 of 22 (50%) patients taking oral antipsychotics. When offered, more LAI-naïve patients expressed neutral (9 of 19 [47%]) rather than favorable (3 of 19 [16%]) or unfavorable (7 of 19 [37%]) responses. Prescribers were most concerned about potentially damaging the therapeutic relationship and side-effects when discussing LAIs while patient resistance was often related to negative feelings about injections. Psychiatrists had some success in overcoming patient objections to LAIs by addressing and decomposing initial resistance. More than half (11 of 19 [58%]) of LAI-naïve patients agreed to start LAI treatment following office visits. Patient-described benefits of LAIs vs orals included perceived rapid symptom improvement and greater overall efficacy.

**Conclusions:**

In this study, many psychiatrists did not offer LAIs and most patients and caregivers were not involved in antipsychotic treatment decision making. Opportunities to increase active patient engagement, address resistances, guide patient drug-formulation selection, and provide better LAI-relevant information for more individualized approaches to treating the patient with schizophrenia were present.

## Background

Schizophrenia can be thought of as a group of chronic disorders that are often neurodevelopmentally based and marked by progressive brain changes, tissue loss (both gray and white matter), and increases in ventricular volume [1] that have been associated with functional impairment [2] and increased hospitalization rates [3]. Long durations of untreated psychosis are associated with more widespread symptomatology, lower quality of life, and may also lead to a lower chance of achieving remission [4,5]. Patients may show decreased responsiveness to treatment following a relapse [6] and increased time to remission may occur with each subsequent relapse [7]. The highest chance of remission or recovery is at the first episode [8] and with continued treatment [9]. These conclusions, along with findings that certain antipsychotics have been associated with attenuation of frequently observed brain grey matter loss [3], suggest that rapid and consistent treatment may help avoid accumulation of permanent disability.

The definition of medication adherence or compliance varies in the literature but has been recently defined as ≥80% of medications taken (over 12 months) and/or <1 week of missed medications (over 3 months) [10]. Adherence is important for effective treatment and relapse prevention. Recent surveys suggest that, on average, experts believe patients with schizophrenia only take 51% to 70% of their prescribed medications [10], with only 40% to 60% of patients remaining adherent to treatment long-term [11]. Even these numbers may be optimistic as adherence statistics are often based on self-reporting due to a lack of available objective and accurate measures [10].

Adherence can be a significant predictor of recovery among schizophrenia patients [12]. Remaining on antipsychotic medication can lead to long-standing remissions and improved quality of life [9]. Nonadherent patients are over 10 times more likely to have a psychotic relapse and 4 times more likely to be hospitalized than adherent patients [13]. Reduced adherence can also complicate treatment assessments based on the inability to discern whether poor outcomes stem from the choice of medications or from failure to take them as prescribed [10]. These detrimental effects on clinical outcomes are also a significant factor on health care burden. In 2005, rehospitalization costs related to antipsychotic nonadherence in the United States (US) ranged from $1.4–$1.8 billion [14]. In one study, nearly 30% of partially or fully nonadherent patients were hospitalized over 1 year compared with 17% of adherent patients [15]. The average length of hospital stay for nonadherent, partially adherent, and adherent patients was 18 days, 30 days, and 9 days, respectively [15].

Factors that contribute to medication compliance include: cognitive deficits, perceived or actual side effects, lack of patient insight, poor efficacy, lack of social support, problems with the therapeutic alliance, cultural or religious beliefs, complexity of daily treatment regimens, and drug abuse among others [10,16]. Environmental factors like unstable living situations, lack of insurance or financial issues, and difficulty with access to treatment can also influence adherence [10].

Long-acting injectable antipsychotics (LAIs), which are administered once every 2 or 4 weeks (depending on the specific drug) rather than daily, are one option to help address nonadherence. LAIs have been associated with both reduced hospitalization rates and care costs [17-19]. Clinical guidelines on LAI usage have been published, recommending that LAIs should not only be used to address nonadherence but also if patients have a basic preference for this formulation––for convenience or any other reason [20-22]. Yet, LAIs continue to be underused and are often reserved for only the most severely affected/nonadherent patients. Despite a low adherence rate with oral medication overall [11], US prescription rates for LAIs have been estimated at only 8% of schizophrenia patients receiving treatment (estimated from the IMS Multinational Integrated Data Analysis System database, Q3 2011–Q2 2012 [e-mail communication to the author from Ray Lansigan of Rosetta–a marketing research organization (ray.lansigan@rosetta.com), February 13, 2013]). In contrast, surveys in the United Kingdom, Belgium, Hong Kong, and Australia have found LAI use to vary between 22% and 36% of patients prescribed an antipsychotic [23-26].

Patient and prescriber perspectives may present obstacles to using LAIs. A recent systematic literature review of publications from June 1999 to the end of February 2008 found that only 1 out of 5 evaluated studies showed positive patient attitudes towards LAIs (the remaining 4 were divided evenly between neutral and negative attitudes) [27]. In comparison, health care professionals (HCPs) had more favorable opinions about LAIs, with 4 of 7 studies reporting positive attitudes [27]. These findings were supported by survey results that showed patients had less favorable views of LAIs than psychiatrists or caregivers [28]. Interestingly, a strong positive relationship was identified between patient attitudes and prior experience with LAIs. Positive perceptions were indicated by 23% of LAI-naïve patients, 45% who had previously received LAIs, and 73% currently taking LAIs [29], suggesting experience with LAIs significantly affects perspectives on treatment with LAIs.

For psychiatrists, there seems to be a positive relationship between level of knowledge about LAIs and attitude toward LAIs [30]. Among psychiatrists, despite a minority expressing negative attitudes such as feeling that LAIs might require coercion or be viewed as old-fashioned or stigmatizing [30], most prescribers expressed favorable attitudes about LAI formulations, particularly for patients with adherence or relapse issues [30]. However, this support does not appear to translate into practice because approximately 9 out of 10 psychiatrists endorse oral formulations over LAIs [28].

This study was designed to examine contemporary actual office visit interactions between patients, caregivers, and prescribers to further understand and characterize the dynamic interaction between prescriber and patient perspectives on the use of LAIs for the treatment of schizophrenia. The overall goal is to facilitate better clinical understanding of the obstacles surrounding use of LAIs and describe successful approaches to offering this formulation option to realize greater success in clinical outcomes and achieving therapeutic goals.

## Methods

### Health care professional/patient recorded conversations

Psychiatrists who worked in a community mental health center (CMHC) were selected from a panel previously identified to participate in ethnographic research (ie, a non-random, self-selected sample). Nurse practitioners (NPs), social workers, and therapists were recruited by telephone. All professional participants completed a screening questionnaire to ensure qualification for the study. HCPs provided study information to patients and Health Insurance Portability and Accountability Act (HIPAA) informed consent was acquired from all study participants. HCP participants were compensated for recording their visits with patients and patients were not compensated during this phase of the research.

Psychiatrists and NPs were included if ≥50% of their practice occurred in a CMHC, they treated patients with schizophrenia, and prescribed LAIs for at least some patients. Other HCPs (eg, social workers) were included if >20% of their time was spent in a CMHC and they worked with patients with schizophrenia. Patients in the study had a primary diagnosis of schizophrenia and were indicated for a change in treatment. Patients with a primary diagnosis other than schizophrenia, those who were non-English speaking, or who could not provide informed consent due to cognitive impairment were excluded.

**Figure 1:**
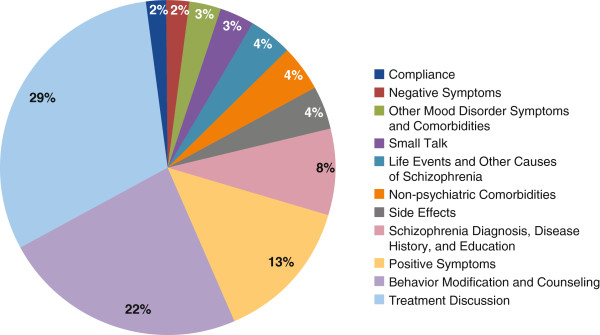
**Topics discussed as percent of prescriber-patient community mental health center office visits (n = 69).** (Total does not equal 100% due to some language/discussion not falling under 1 of topics).

Conversations between patients or their caregivers and HCPs from across the US were recorded during treatment visits between August 2011 and February 2012. Conversations were transcribed and analyzed. Discussions were categorized according to 11 main predetermined topics occurring during a typical CMHC visit for patients with schizophrenia (Figure 1).

A total of 2–4 team members (consisting of 2 medical information researchers and 2 linguists/cultural anthropologists) concurrently analyzed the recorded conversations by reading and listening to each dialogue. Analyses were descriptive and qualitative. Qualitative and linguistic analysis was conducted utilizing a mix of analytical methods including “constant comparison” [31]. Constant comparison is a method that structures analysis of conversational dynamics, lexicon, themes, and meaning across a corpus of data and within cohorts. In constant comparison, the analyst selects 2 data points (here, HCP-patient/caregiver conversations) and analyzes first one side and then the other, for conversational dynamics, lexicon, emergent themes, and essential meanings. Then, the second conversation is analyzed in the same way, noting similarities and differences between the 2 conversations in a spreadsheet and within the texts themselves, such that a rich log of findings and comparative points is constructed and maintained. The analyst then proceeds with a third data point (ie, one side of the next conversation pair), comparing findings from that text to the prior 2 texts and notes any differences or similarities among all 3. Then the analyst compares the fourth data point to findings from analysis of the first 3. This proceeds across the corpus of data within a cohort. Constant comparison is then conducted in other cohorts in this manner. Findings from all cohorts are then compared and contrasted to identify essential differences between and among cohorts.

### Further telephone in-depth interviews

Further telephone in-depth interviews (TDIs) with psychiatrists (n = 8), patients (n = 12, monthly × 3 months), and caregivers (n = 4) also were conducted to gather follow-up information on LAI discussion, prescription, or use. During TDIs with psychiatrists, audio clips from actual conversations from the patient meeting were played back to gain deeper insight into thought processes during the conversations.

### In-person community mental health center observation

Separately, 4 CMHCs (located in Chicago, IL; Atlanta, GA; Warren, OH; and St. Petersburg, FL) were recruited for in-person observations that took place between December 2011 and February 2012. CMHC psychiatrists provided study information to patients and patients then had to contact study investigators if interested in participating. Informed consent was acquired from all participants in the study. Professional participant and patient/caregiver information collected was made anonymous prior to analysis. The study protocol was approved by the independent New England Investigational Review Board. HCPs were not compensated for their participation, however, the CMHC was provided with a $1500 donation. Patients received a $25 local gift card for participating in the in-person observations.

The research was carried out by teams of 1–2 trained anthropologists. Anthropology researchers spent a few hours at home with some patients prior to their CMHC visit, discussing each patient’s disease and treatment experiences, then followed the patient to the CMHC and observed any meetings with the psychiatrist or nurse, the patient receiving an injection, and any other interactions within the clinic. A total of 15 patient in-home visits, with 10 patients receiving LAIs and 5 receiving oral medications (12 men and 3 women) were conducted.

## Results

### Patient and prescriber (or other health care professional) conversations

Prescriber conversations (n = 60 with 14 psychiatrists; n = 9 with 2 NPs) averaged 11.5 minutes in total duration. Duration of interaction varied individually by type of HCP, averaging 12 minutes with psychiatrists, 9 minutes with NPs, and 16.6 minutes with social workers or therapists (4 conversations from 2 social workers and 2 therapists). Conversations between all types of HCPs and patients or caregivers comprised 2 phases: assessment and decision, which each covered approximately 70% and 30% of conversation time, respectively. During the assessment phase of the conversation, patients and/or caregivers dominated the conversation and then generally yielded to HCPs for the decision phase.

**Figure 2:**
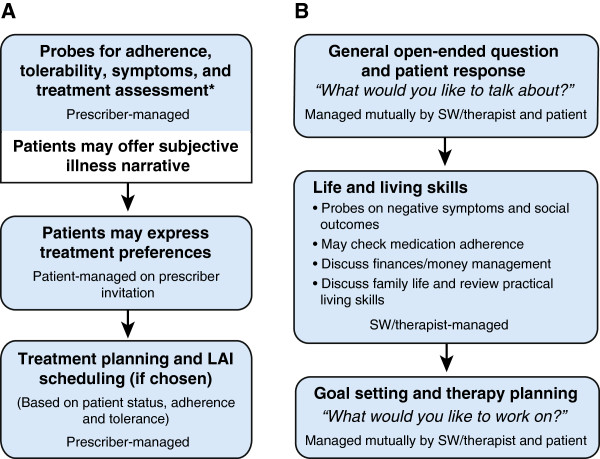
Observed conversation flow between: A. patients and prescribers (n = 69); B. patients and social workers or therapists (n = 4).

Multiple treatment goals were pursued for patients with schizophrenia and were addressed differently by each type of HCP. Social workers and therapists used open-ended questions (“What would you like to talk about?” “What would you like to work on?”) and primarily focused on issues like social wellness and means of achieving daily structure, like work or school. Patients or caregivers sometimes discussed medications and compliance during these sessions but were not explicitly focused on this topic. Prescribers typically used a scripted check-list approach to ensure assessment for positive symptoms, deviating only if positive symptoms were detected and required further investigation. Figure 1 shows a breakdown of the types of topics discussed between prescribers and patients. Treatment discussion and behavior modification/counseling occupied just over 50% of the prescriber-patient visit. Psychiatrists and NPs spent a similar amount of time on treatment discussion while the time focused on behavior modification/counseling is attributed more to psychiatrists (25% vs 1% for NPs). Discussion on compliance occupied only 2% of the prescriber-patient visit. During conversations about medications, prescribers asked simple, direct questions when probing for patient medication compliance (“Are you taking your medications?” “Did you take any medicine last night…?”). Prescribers used direct and logical strategies when probing adherence but could become more authoritative upon discovering noncompliance. Overall conversation flow generally started with prescribers probing for compliance, symptoms, and assessing treatment, which may have included a subjective illness narrative by the patient. The second phase was led by patients where they could express any treatment preferences. The final phase consisted of prescriber-led treatment planning and LAI scheduling (if selected) based on learnings from the earlier phases of the conversation (Figure 2).

### Health care professional and patient characteristics

A total of 20 unique HCPs (psychiatrists, n = 14; nurse practitioners, n = 2; case/social workers, n = 2; therapists, n = 2) from 16 unique institutions across the United States participated in the study.

Psychiatrists (n = 14) and their patients (n = 60) provided the most complete set of information for the study including recorded conversations, TDIs, and in-person CMHC observations. Psychiatrists’ patients were being treated with oral antipsychotics (n = 22) or LAIs (n = 38). Psychiatrist could be treating individual patients with LAI or oral antipsychotic medications. Psychiatrist and patient characteristics by type of treatment are listed in Table 1. Median years in practice for psychiatrists treating LAI patients and those treating patients on orals were 25 and 18 years, respectively. Patients receiving oral treatment were predominantly female (59%) and LAI patients were mostly male (53%). The majority of patients across both groups (58% to 64%) were initially diagnosed with schizophrenia >10 years prior to the study. More than one fourth (27% to 29%) of the sample were diagnosed within 5 years.

**Table 1:** Psychiatrist and patient characteristics by type of treatment

**Characteristic**	**Patients on oral antipsychotics (n = 22)**	**Patients on LAIs (n = 38)**
**Treating psychiatrists**	**9**	**11**
Years in practice, mean, (SD)	16.1 (8.5)	18.5 (8.2)
Years in practice, median	18	25
**Patients**		
Men, n (%)	9 (41)	20 (53)
Women, n (%)	13 (59)	18 (47)
Age, y, mean	45.7	38.9
**Time since diagnosis, n (%)**		
≥10 y	14 (64)	22 (58)
>5–10 y	1 (4.5)	5 (13)
>1–5 y	6 (27)	10 (26)
6–12 months	NA	1 (3)
Unknown	1 (4.5)	NA
**Previous psychiatric hospitalization, n (%)**	17 (77)	33 (87)
**Number of previous injections for patients currently on LAI, mean**	NA	2.69

LAI, long-acting injectable antipsychotic; SD, standard deviation; y, years.

### Treatment decisions and conversations on long-acting injectable antipsychotics

Psychiatrists made antipsychotic treatment decisions without patient or caregiver input during 40 of 60 (67%) conversations. Patients with less severe impairment were more likely to be involved in treatment decisions (conversations with 13 of 36 [36%] mild or moderate patients vs 7 of 24 [29%] severe patients). Involvement in treatment decisions was greater when discussing LAIs: 15 of 60 (25%) with patients/ caregivers vs decisions about oral antipsychotics, 5 of 60 (8%). However, there were no discussions of LAIs by psychiatrists in 11 of 22 (50%) patients taking oral antipsychotics (Table 2), despite the fact that participating patients were indicated for a change in treatment. Overall, only 6 of the 60 conversations (10%) involved patients actively making an antipsychotic treatment decision.

**Table 2:** Patient-psychiatrist conversations about LAIs

**LAI patient status/conversations, n (%)**	**Patients on oral antipsychotics**	**Patients on LAIs**
	**(n = 22)**	**(n = 38)**
Previously treated with LAI^a^	3 (14)	NA
Discussion of oral treatment	3	NA
Discussion of LAI treatment	3	NA
Oral with discussion of LAI	8 (36)	NA
Discussion of oral treatment	8	NA
Discussion of LAI treatment	8	NA
No discussion of LAI	11 (50)	NA
Discussion of oral treatment	11	NA
Discussion of LAI treatment	0	NA
LAI discontinuation	NA	1 (3)
Discussion of oral treatment	NA	1
Discussion of LAI treatment	NA	1
LAI restart	NA	2 (5)
Discussion of oral treatment	NA	2
Discussion of LAI treatment	NA	2
LAI-to-LAI switch	NA	3 (8)
Discussion of oral treatment	NA	3
Discussion of LAI treatment	NA	3
New start on LAI	NA	11 (29)
Discussion of oral treatment	NA	9
Discussion of LAI treatment	NA	11
LAI continuation	NA	21 (55)
Discussion of oral treatment	NA	13
Discussion of LAI treatment	NA	21
**LAIs used in treatment, n (%)**		
First-generation LAIs	NA	8 (21)
Second-generation LAIs	NA	30 (79)

^**a**^ Discussion of history of LAI use, not restarting LAI treatment.

LAI, long-acting injectable antipsychotic.

**Figure 3:**
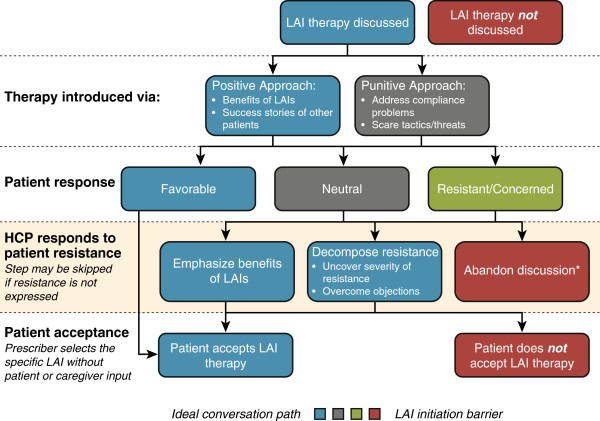
Observed conversation decision tree for prescriber interactions with patients regarding initiation of long-acting injectable antipsychotics.

The conversation flow around introducing LAIs typically followed a number of steps that could be terminated by the prescriber or patient at several decision points (Figure 3). More than half (11 of 19 [58%]) of LAI-naïve patients offered LAIs by their psychiatrists agreed to start treatment although just three of those who agreed (3 of 11 [27%]) verbalized favorable responses to an LAI (Table 3). Adherence benefit was the major verbalized reason for accepting an LAI offer and fear of needles was most common for refusals. Almost half of patients offered an LAI were neutral or passive in the decision. During these conversations, a variety of techniques were used to encourage patient acceptance of LAI treatment, including: personal gain to the patient (“…not having to worry about where your pills are…”); sharing other patients’ experience (“Sometimes, patients think that this is easier …”); or occasionally use of fear tactics (“…those voices, those paranoid thoughts are all going to come back. It’s just a matter of time…I can guarantee…that it will happen”). When caregivers were present they were supportive of psychiatrists’ choice of LAIs. However, this was only examined with a small sample, as caregivers were only present in 3 of 19 (16%) discussions with LAI-naïve patients.

**Table 3:** Patient reactions to psychiatrist’s offer of long-acting injectable antipsychotic treatment

**LAI-naïve patient reactions to**	**n (%)**	**Prescribed a LAI, n**
**LAI offer (n = 19)**		**(% of reaction category)**
Favorable	3 (16)	3 (100)
Neutral/passive	9 (47)	6 (67)
Unfavorable/concerned	7 (37)	2 (29)
**Verbalized patient reasons for LAI acceptance (favorable)**^**a**^**, n**		
Adherence benefits		3
Extended/consistent efficacy		1
Lessen oral pill burden		1
**Verbalized patient reasons for LAI refusal (unfavorable)**^**b**^**, n**		
Fear of needles		3
Dosing logistics/administration		2
Side effect concerns		2
Unclear		1

^a^Two patients cited 1 other benefit in addition to adherence.

^b^One patient cited 2 reasons for refusal of LAI.

LAI, long-acting injectable antipsychotic.

If the decision was made to initiate LAI treatment, psychiatrists selected the specific LAI to prescribe with minimal patient input. Only 1 specific LAI was discussed in most of the “new start” conversations (7 of 11 [64%]). Patients and caregivers confirmed in TDIs that LAI selection had been made without their input and they were generally uninformed about choices (“I don’t think there are that many choices with [the] shot.”).

When the discussion about initiating LAIs was abandoned by prescribers, the main reason stated was to preserve a healthy, trusting therapeutic relationship with the patient rather than risk being perceived as coercive. Second, prescribers felt it was important to allow patients to retain autonomy to create treatment “buy-in”. Third, prescribers felt that patients who initially rejected LAIs could become more receptive over time and chose to reintroduce the idea at a later date.

### Barriers to initiation of long-acting injectable antipsychotics

Patient obstacles to LAI use emerged as fear or hesitation about the injections. These perceived fears most consistently impeded LAI prescription choice. Patients who expressed strong concern about injections often did so repeatedly in the conversation (“I never did injections, I don’t like needles…They freak me out, they scare me, they hurt and I don’t like them.”). Despite multiple tactics attempted by psychiatrists, the persistent refusal of patients who expressed a strong fear and concern about injections avoided use of LAIs. Of the 7 of 19 (37%) LAI-naïve patients who responded unfavorably to an LAI offer from a prescriber, only 2 of 7 (29%) received an LAI.

Another barrier to LAI use was the lack of patient insight into the disease and treatment (inability to reason regarding symptoms and treatment options). Patients generalized the negative treatment experience with a single LAI to the entire class of LAIs, even if there was a clear distinction between that past experience and currently available options.

Other examples of potential barriers to LAIs reported by individual patients included the requirement to go to the CMHC to receive injections and wishing treatment with medication was more effective. It is unknown to what extent these reasons ultimately prevented patients from receiving injections. In TDIs, the cost of medication as a barrier was reported in a very small number of cases as most patients received state or federal assistance (8 of 12 [67%]) or held private insurance (3 of 12 [25%]).

Among prescribers, possible side effects were among the chief concerns for LAI usage, specifically, with the long-acting effects of this administration method because treatment cannot be withdrawn rapidly if side effects suddenly occur. Despite this concern, conversations about side effects were rarely initiated by psychiatrists, typically being left for the patient to initiate. Even when specific side effects were explored (eg, with the use of general questions such as, “Have you been sleeping okay?” or “Is your appetite okay?”), they were not always directly attributed to the medication. Similar to psychiatrists, NPs tended to use very general questions about side effects, and during conversations did not always differentiate among side effects of LAIs vs. oral medications. However, LAI treatment changes were rare–only 4 of the 38 patients treated by psychiatrists switched or discontinued LAI treatment: 1 discontinued due to restless legs and other unspecified side effects, while 3 switches to a different LAI occurred due to fatigue/grogginess (n = 1) and high prolactin levels (n = 2). It should also be noted that psychiatrists’ patients on an LAI at time of study had previously received an average of 2.7 injections, suggesting they had probably only recently begun treatment.

### Overcoming barriers to treatment with long-acting injectable antipsychotics

Prescribers were most successful in overcoming patient objections to LAIs by decomposing resistance to uncover the severity of resistance and investigate beyond the initially stated problem to address the root issue. Prescribers used several other logic-based approaches to overcome barriers during discussions. Emphasizing the benefits of newer LAIs, like the use of smaller needles with certain injections, or better therapeutic effects with LAIs than their oral counterparts helped patients commence LAI treatment. Empowering patients during the decision (“…just commit to one month of medicine, that’s all, just one shot. If it’s a disaster we’ll switch gears”), emphasizing convenience (“one shot and we can pretty much minimize all medication”), or showing patients the needle and talking to the nurse were also successful approaches. In terms of decomposing resistance, one particular example included a patient who claimed to have a fear of needles, yet was actually resistant due to a 20-lb weight gain with a previous LAI. Digging deeper into the objection was successful. (Patient: “I actually, I have problems with needles. …last year, [my doctor was] giving me those [specific LAI medication] shots and it made me gain 20 pounds in 1 week”, Psychiatrist: “So it wasn’t the injection, per se, it was the side effect of the medicine. There is a different injection we can use. This is a once a month and I have several clients on it who have not gained weight.”).

### Determinants of continued use of long-acting injectable antipsychotics

Twelve patients who received LAIs participated in TDIs; 9 of 12 (75%) believed they had improved over the 3 months during which the interviews occurred and attributed their success mainly to their LAI treatment. No patients reported a worsening condition. Individual patients mentioned benefits of LAIs that included: improvements in symptoms, better concentration, attention, alertness, and a more positive outlook. Patients also mentioned the medication working “faster” and “better” than oral formulations. Generally, strong support systems and an absence of barriers kept patients adherent to the LAI schedule. As with oral medications, stable home environments, involved family members, friends, and other such caregivers, and case workers helped contribute to adherence. Addressing logistical issues like transportation services to enable patients to reach a CMHC for treatment also encouraged LAI usage and adherence.

Information seeking and communication about LAIs may have contributed as another determinant for LAI usage, particularly by using the Internet. Most patients and caregivers reported using the Internet most commonly to search for information about schizophrenia, with most activity occurring around the time of diagnosis. Additionally, a few patients and caregivers reported social media sites (eg, blogs, message boards, chat rooms) to be the most useful source of disease information.

During TDIs, LAI patients reported willingness to share positive experiences with other patients including convenience, efficacy, and concerns about the injections/needles. The above may serve as future assistance for other patients considering LAI treatment.

## Discussion

This study provided information to characterize the process and content of prescriber-patient interactions, perspectives around treatment with LAI antipsychotics and prescriber strategies to overcome barriers. Although many psychiatrists do not routinely offer LAIs to their patients and do not involve patients in antipsychotic treatment decision making, many patients are willing to start LAIs due to their neutral perspectives on the matter. Decision points around starting/restarting or not starting LAIs in this study seemed to be influenced by the pre-established beliefs concerning LAIs for both prescribers and patients; the majority of prescribers were concerned about damaging the therapeutic relationship and side effects. Some patients have negative feelings about the injection, however, more LAI-naïve patients expressed neutral rather than favorable or unfavorable responses when offered LAIs and this presents an opportunity for guiding decision making and a deeper dialogue.

One quarter (15 of 60 [25%]) of patient-prescriber conversations were focused on LAIs, whereas only 8% (5 of 60) were focused on orals. This finding suggests that prescribers seemed to welcome patient involvement in the LAI treatment decisions as much, if not more, than oral treatment decisions. Although a minority of patients was actively involved in final treatment decisions, there seems to be room for more active patient engagement to move past resistance through additional assessment of patient concerns and provision of relevant information. When patients play active roles by asking questions and exchanging information [32], they may feel more in control of their treatment and, therefore, be more receptive to LAIs. Prescribers may also need to allocate more time to discussion of adherence to the patients’ current medication as another possible method of opening conversation on use of LAIs. In the current study, discussion of adherence to current medication occupied only 2% of the prescriber-patient visit. However, there may be limited time for prescribers to discuss treatment options if the average time spent with the patient is 12 minutes or less as was found in this study.

The current study also seems to support findings that increasing knowledge of the provider [30] and information on positive LAI experiences of the patient [29] may improve attitudes about LAIs and facilitate the prescribing process. Providing increased information on simplicity, safety, and tolerability of LAIs, and feedback of positive experiences/outcomes could be helpful to reduce resistance of both prescribers and patients.

Despite guidelines suggesting that LAIs be used for convenience or patient preference (and not exclusively to address adherence) [20-22], there was little direct evidence in the current study that LAIs were offered with convenience as the primary determinant. Most prescribers still appeared to consider LAIs best for patients with current or potential nonadherence issues with oral formulations, citing convenience merely as a tactic to encourage patients to accept LAIs. Interestingly, a recent survey showed that 64% of LAI prescriptions were based on patient request, compared with 43% for nonadherence [28]. The survey also indicated psychiatrists would prescribe LAIs more frequently if patients would accept this formulation [28]; the disconnect is evident in that nearly two-thirds of LAI-naïve patients were not even informed of this option [28]. Increased awareness among both doctors and patients of the option to prescribe/receive LAIs for reasons other than adherence may help inform treatment decisions.

It should also be noted that patients in the current study using oral medications were about 7 years older (mean age: 45.7 years) than those taking LAIs (38.9 years). In contrast, the Patel et al. [33] study of patient preferences and attitudes towards LAIs found that patients on LAIs were significantly older than patients on oral antipsychotics. It is not known how age and other patient characteristics impact perspectives on treatment with LAIs. Other patient characteristics such as the effect of patient cultural perspectives were not investigated and would be worth further study.

Only 4 caregivers were surveyed in this study. However, results here and in the literature suggest caregiver involvement facilitates LAI usage. During the current study, caregivers were only present in 16% (3 of 19) of conversations between psychiatrists and LAI-naïve patients, but in each case encouraged the use of LAIs. Overall, there has been relatively little investigation on caregiver attitudes about LAIs compared with those of HCPs and patients. A PubMed search in October 2012 using the string *long-acting injectable antipsychotics attitudes* yielded 35 articles, whereas adding the terms *caregivers* or *relatives* yielded 2 and 4 articles, respectively. One article indicated stronger support from caregivers than from patients for the potential advantages of LAIs [28].

Despite the valuable information gained in the current study, there are some obvious limitations. The information obtained was subjective, difficult to quantify, and from a small, non-random sample that included few caregivers. Accordingly, there were no formal statistical analyses performed to compare different approaches, attitudes, or usage of terms during conversations, and any direct effects on LAI usage patterns. The rate at which HCPs prescribed oral and LAI antipsychotic formulations prior to the study was not captured and it is unknown how their prescribing trends compared with national rates. In addition, it is possible that ethnic/racial or primary language differences between patients and prescribers may have influenced patients’ perceptions and reactions during conversations; however, this information was not captured. A larger scale study with quantifiable variables and statistical power would be useful to address these limitations and to objectively identify approaches to help both prescribers and patients make more informed treatment decisions.

## Conclusions

Although some prescriber and patient perspectives on LAIs have been described in the literature, the additional dynamic understanding of prescriber-patient interactions gained here by analyzing actual conversations provided further insights. The prescriber-patient visit presents opportunities for deeper dialogue on the use of LAIs and more patient and caregiver involvement in decisions about treatment options and goals. Increasing knowledge about LAIs and the process of initiating treatment, for the prescriber and patient, may facilitate use of LAIs. For patients, this can occur when prescribers actively engage the patient to move past initial resistance to LAIs. However, more needs to be known about the effects of patient characteristics and caregiver involvement on treatment decisions. The process, perspectives, barriers, and approaches identified here may help inform treatment decisions when prescribing LAIs and stimulate additional research leading to more effective individual approaches to treatment for the patient with schizophrenia.

## Competing interests

SP: Received grant funding from Astra-Zeneca, Bioline, Bristol-Myers Squibb, Sunovion, Forest Laboratories, Janssen Pharmaceutica, Merck, Novartis, Otsuka, Pfizer Inc., Solvay Pharmaceuticals, Roche, and Vanda Pharmaceuticals Inc. He also serves as an investigator for Otsuka, and as a consultant/advisory board member for the American Psychiatric Association, AstraZeneca, Bioline, Bristol-Myers Squibb, Concert, Cortex, Sunovion, Janssen Lundbeck, Merck, Novartis, Organon, Otsuka, Pfizer Inc., Roche, Schering Plough, Takeda, and Vanda Pharmaceuticals Inc. SP serves on the Speakers’ Bureau for Lundbeck, Merck, Novartis, Pfizer Inc., and Sunovion. RB: Served as a consultant for Otsuka. DZ and GL are employees of Otsuka America Pharmaceuticals Inc.

## Authors’ contributions

SP was involved in all aspects of the analysis and interpretation of the data. He was fully involved in drafting the manuscript and revising it critically for intellectual content. RB contributed to design of the study, and was involved in analysis and interpretation of the data, drafting the manuscript and revising it critically for intellectual content. DZ contributed to study design and data acquisition, interpretation of the data, drafting the manuscript and revising it critically for intellectual content. GL was involved in analysis and interpretation of the data, helped in drafting the manuscript and revising it critically for intellectual content. All authors read and approved the final manuscript.

## Pre-publication history

The pre-publication history for this paper can be accessed here:

http://www.biomedcentral.com/1471-244X/13/261/prepub
